# Correlates of Transitions in Food Insecurity Status during the Early Stages of the COVID-19 Pandemic among Ethnically Diverse Households in Central Texas

**DOI:** 10.3390/nu13082597

**Published:** 2021-07-28

**Authors:** Kathryn M. Janda, Nalini Ranjit, Deborah Salvo, Aida Nielsen, Pablo Lemoine, Joy Casnovsky, Alexandra van den Berg

**Affiliations:** 1 UTHealth School of Public Health, Austin, TX 78701, USA; Nalini.Ranjit@uth.tmc.edu (N.R.); Aida.Alibegovic@uth.tmc.edu (A.N.); Alexandra.E.VanDenBerg@uth.tmc.edu (A.v.d.B.); 2 Michael and Susan Dell Center for Healthy Living, Austin, TX 78701, USA; 3 Prevention Research Center, Brown School, Washington University in St. Louis, St. Louis, MO 63130, USA; dsalvo@wustl.edu; 4 Centro Nacional de Consultoría, Bogotá 110221, Colombia; plemoine@cnccol.com; 5 Sustainable Food Center, Austin, TX 78702, USA; joy@sustainablefoodcenter.org

**Keywords:** food insecurity, disparities, food access, food availability, COVID-19

## Abstract

Food insecurity increased substantially in the USA during the early stages of the 2020 COVID-19 pandemic. The purpose of this study was to identify potential sociodemographic and food access-related factors that were associated with continuing or transitioning into food insecurity in a diverse population. An electronic survey was completed by 367 households living in low-income communities in Central Texas during June–July 2020. Multinomial logistic regression models were developed to examine the associations among food insecurity transitions during COVID-19 and various sociodemographic and food access-related factors, including race/ethnicity, children in the household, loss of employment/wages, language, and issues with food availability, accessibility, affordability, and stability during the pandemic. Sociodemographic and food access-related factors associated with staying or becoming newly food insecure were similar but not identical. Having children in the household, changes in employment/wages, changing shopping location due to food availability, accessibility and/or affordability issues, issues with food availability, and stability of food supply were associated with becoming newly food insecure and staying food insecure during the pandemic. Identifying as Latino and/or Black was associated with staying food insecure during COVID-19. These findings suggest that the COVID-19 pandemic did not create new food insecurity disparities. Rather, the pandemic exacerbated pre-existing disparities.

## 1. Introduction

Food insecurity, defined as a lack of consistent access to sufficient food for an active and healthy life, is a critical social determinant of health and a major contributor to health inequalities [1,2,3,4]. Food insecurity is known to be related to unhealthy dietary behaviors [4], and to many diet-related chronic conditions including obesity [5], type 2 diabetes mellitus [6,7], and heart disease [8,9]. Food insecurity is estimated to cost the United States (USA) over 182 billion USD per year, primarily due to increased health care costs [10]. Increases in food insecurity during COVID-19 [11,12,13], thus, have implications for society as a whole [14], yet little is known about who is particularly vulnerable to staying food insecure or becoming food insecure. 

### 1.1. Food Insecurity and Food Access

Conceptually, food insecurity is comprised of four components: availability, access, utilization, and stability over time [15,16]. Availability is defined as physical availability of food in one’s country or community; access is comprised of several components including physical (geographic) and economic accessibility, cultural acceptability, and safety of consumption; utilization refers to individual level consumption and absorption; and stability is a cross-cutting dimension that requires that availability, access, and utilization are present at all times [16,17]. While there is a wealth of research about the roles of stability and utilization in low- and middle-income countries [15,16,18], the majority of food insecurity studies conducted in the United States have focused on the role of food access, specifically geographic and economic food access [17,19,20]. Thus, studies should also be conducted that consider food access factors in the examination of food insecurity. 

### 1.2. Food Insecurity and Food Access Disparities 

Food insecurity does not occur in isolation and is often the byproduct of poverty or economic disadvantage [21,22,23]. Historically, people of color and low-income households are more likely to be food insecure than people who are non-Hispanic white and people who live in higher income households [24,25,26]. Communities of color and low-income communities are more likely to have limited geographic food access, meaning that they typically do not have geographically and/or economically accessible healthy food retail options in their neighborhoods and have to travel farther to access food [27,28,29]. These disparities are evident in Central Texas, where Eastern Travis County has historically had a larger population of Black and Latino households, lower median household income, fewer healthy food retail opportunities, and a higher prevalence of food insecurity as compared with Western Travis County [30,31,32,33].

### 1.3. Food Insecurity during the COVID-19 Pandemic

During the COVID-19 pandemic, the relatively sudden and compressed onset of high unemployment rates, economic downturn, stay at home orders, school closures (and consequently the reduced offering of school nutrition programs), closure/limited hours for food retail, and social distancing policies have had a particularly dramatic impact on food insecurity [12,13,28,34,35,36,37,38]. Early in the pandemic, Feeding America projected that up to an additional 17 million people could soon become food insecure [39], adding to the nearly 13.7 million that were already insecure in 2019, for a total of 23.5%. These projections were underestimations when compared to research done at Northwestern University, which estimated that food insecurity prevalence increased to 25.5% during the COVID-19 pandemic [11]. Paralleling national-level trends, the prevalence of food insecurity prevalence in Texas doubled from 2018 to April–June 2020 (from 14 to 28% respectively) [11]. 

However, the aforementioned study focused on the prevalence of food insecurity in general, and did not differentiate between those who became newly food insecure due to the COVID-19 pandemic (i.e., incidence of food insecurity) versus those who were consistently food insecure before and during the pandemic. To our knowledge, only one study has differentiated between staying food insecure and the incidence of becoming newly food insecure during the COVID-19 pandemic; however, this study was limited to a sample that was racially/ethnically homogenous, and non-Hispanic white [13]. Therefore, there remains a need to differentiate between those who stayed food insecure and those who became newly food insecure during the pandemic and to investigate the potential role of sociodemographic and food access-related factors within racially/ethnically diverse communities. The purpose of this study was to use cross-sectional survey data to examine and identify sociodemographic and food access-related factors differentiating households that became newly food insecure during the COVID-19 pandemic, households that were consistently food insecure before and during the pandemic, and households who stayed consistently food secure.

## 2. Materials and Methods

### 2.1. Study Design and Sample 

The data presented in this manuscript are a cross-sectional analysis of survey data with a sample that was part of a larger longitudinal cohort study which started in 2018. The original cohort sample was recruited in October 2018–March 2019 as part of a larger evaluation of a healthy food retail initiative being implemented in Eastern Travis County. The original cohort sample was predominantly Latino (54.41%), lower income (52.62%), and resided in lower income zip codes with historically limited geographic food access. The sample utilized for this study included original cohort members, as well as referrals by the original cohort that were recruited through a snowballing method. During the months of June–July 2020, a total of 688 individuals (394 members of the original cohort + 294 referrals) were invited to take part in an online survey via email or a text message sent through the Qualtrics’ online survey software. Each respondent who completed a survey received a 20 USD gift card and an additional 5 USD gift card if the person they referred also completed a survey. The final sample included a total of 367 people, with 242 original cohort members and 125 referrals; however, analyses were not stratified by recruitment type due to cell sample sizes required for multinomial logistic regression. The survey centered around the COVID-19 pandemic and sought to obtain data on respondents’ experiences with food insecurity, food access, perceived food availability, demographic characteristics, and other variables (not included in this analysis). This study was approved by the UTHealth School of Public Health Institutional Review Board (IRB HSC-SPH-18-0233).

### 2.2. Variables

#### 2.2.1. Outcome Variable: Household Food Insecurity Status during COVID-19

The primary outcome variable for these analyses was household food insecurity status during the COVID-19 pandemic. Household food insecurity was determined by utilizing a two-item food insecurity screener which has been used to classify people dichotomously as food secure and food insecure [40,41,42]. The two-item screener has been validated in a variety of clinical and non-clinical contexts as well as low-income settings [40,41,42]. The screener includes the following two questions: (1) “We (I) worried whether our food would run out before we (I) got money to buy more”, and (2) “The food that we (I) bought just didn’t last and we (I) didn’t have money to get more”. Given that this was studied by employing a cross-sectional study design, the screener was utilized twice in the survey with prompts referring to different specific time points, i.e., before COVID-19, and now/during COVID-19. Participants were classified as “newly food insecure” if their responses to the two-item screener with the “before COVID-19” prompt placed them in the “food secure” category, and their responses to the two-item screener with the “now/during COVID-19” prompt placed them in the “food insecure” category. The resulting variable had three categories: consistently food secure before and during the COVID-19 pandemic, newly food secure during the COVID-19 pandemic, and consistently food insecure before and during the COVID-19 pandemic. 

#### 2.2.2. Sociodemographic Variables

Previous studies in the literature have pointed to a variety of sociodemographic factors associated with food insecurity, including race/ethnicity, children in the household, and employment status. Accordingly, these characteristics, self-reported in the survey, were included in these analyses. Self-identified race/ethnicity was collapsed into a binary variable, Latino and/or Black, and White. The number of children in the household was dichotomized as no children in the household, or one or more children in the household. Employment status and wage changes due to COVID-19 were self-reported in the survey, and collapsed to result in a binary variable: loss of wages and/or employment due to COVID-19, or no change in employment status or wages during the pandemic. A binary variable was also created for language spoken at home, and categorized according to whether the respondent spoke Spanish at home, or English and/or another non-Spanish language at home. Categorization and collapsing variables across categories were to maintain sufficient cell sample sizes, necessary for multinomial logistic regression analyses and face validity.

#### 2.2.3. Food Access-Related Variables

Food access-related factors were also self-reported in the survey in various ways. The variables included on the survey were changes in shopping behavior due to food access-related issues, issues with food availability, perceptions of food prices (affordability), and stability of food in the home. Changes in shopping behavior were measured by asking participants if they changed their usual shopping location during the early stages of the COVID-19 pandemic due to each of several reasons including: usual stores not having the food that was needed (availability), other stores were cheaper than their usual store (affordability), if other stores were closer than their usual store (accessibility), and other options. Due to sample size constraints, to ensure sufficient cell sample sizes necessary for multinomial logistic regression analyses, a binary variable was developed based upon if a participant answered “yes” for issues with availability, affordability, and/or accessibility. 

Respondents were also queried about issues they experienced in finding their normally purchased groceries during the COVID-19 pandemic in three categories: having little to no difficulty, sometimes having difficulty, or always having difficulty. Due to sample size constraints, to ensure sufficient cell sample sizes necessary for multinomial logistic regression analyses and face validity these categories were collapsed into two, i.e., to create a binary variable reporting if they sometimes/always had issues finding their normally purchased groceries during COVID-19 and having little to no difficulty finding their normally purchased groceries during the pandemic. 

Perceptions about food prices were self-reported in the survey and were reported in three categories: food prices increased since the start of the pandemic (i.e., from March to May/June 2020), food prices stayed the same, and food prices decreased since the start of the COVID-19 pandemic. These categories were collapsed to two categories, i.e., reporting a perceived increase in food prices during the pandemic or reporting a perceived decrease or no change in food prices during the COVID-19 pandemic to obtain sufficient cell sample sizes necessary for multinomial logistic regression analyses and face validity. Stability of the food supply in the household was developed from a question that asked respondents how many days of food they had in their home to feed their household if they were unable to go out to buy more. This was dichotomized into having less than a week of food in their home, and having a week or more of food in the home. 

### 2.3. Analyses

Statistical analyses were performed from November 2020 to May 2021 utilizing Stata version 15 (StataCorp Version 15, 2017 College Station, TX, USA). Descriptive statistics included frequencies and percentages were calculated for each categorical variable and indicator. Unadjusted and adjusted multinomial logistic regression models were run to examine how sociodemographic factors of race/ethnicity, presence of children in the household, loss of employment/wages due to the COVID-19 pandemic, language spoken at home, and food access related-factors were associated with food insecurity transitions (consistently food secure, becoming newly food insecure, and staying food insecure during the COVID-19 pandemic). The adjusted multinomial logistic regression model was adjusted for race/ethnicity, presence of children in the household, loss of employment/wages due to COVID-19, language spoken at home, and the aforementioned food access-related factors. 

## 3. Results

### 3.1. Sample and Descriptive Statistics

The total sample included 367 participants (242 original cohort members + 125 referrals from existing cohort members) who completed the COVID-19 cross-sectional survey in June/July 2020. Descriptive statistics of the sociodemographic and food access-related factors of the sample by food insecurity status are presented in Table 1. Among the full sample, the majority of respondents identified as Latino and/or Black (64.69%), had children in the household (60.74%), almost half experienced a change in employment or wages during the COVID-19 pandemic (44.84%), and almost half spoke Spanish in the home (42.35%). Additionally, nearly half of the participants in the full sample reported changing their normal shopping location due to food access-related issues (44.35%). In addition, over half of the participants reported sometimes or always having issues with food availability (52.26%), perceived increased food prices (68.93%), and having less than a week of food for the household at the time of the survey (66.01%).

There were notable differences in the sociodemographic attributes by food insecurity status. Newly food insecure and consistently food insecure respondents were more likely to identify as Latino and/or Black, more likely to have children in the household, more likely to have lost a job or wages during COVID-19, and more likely to speak Spanish at home than their consistently food secure counterparts. Additionally, consistently food insecure respondents had higher response rates of identifying as Latino and/or Black (90.08%), having children in the household (78.86%) and speaking Spanish at home (71.90%) than the newly food insecure respondents (71.43%, 69.70%, and 58.06% respectively).

There were also differences in experiencing issues with food access during the COVID-19 pandemic across food insecurity transition categories. Newly food insecure and consistently food insecure respondents were more likely to report changing their usual shopping location during the COVID-19 pandemic due to food access-related issues, food availability issues, perceived increases in food prices, and having less than a week of food for the household in their home at the time of the survey. For all of these food access-related factors, newly food insecure and consistently food secure respondents had similar response patterns.

### 3.2. Food Insecurity Status during COVID-19 and Sociodemographic and Food Access-Related Factors

The findings from the multinomial logistic regression models examining the relationship between food insecurity status during COVID-19 and sociodemographic and food access-related factors are shown in Table 2. In the adjusted model, various sociodemographic factors were associated with becoming newly food insecure during the COVID-19 pandemic. Households with children had 3.26 times greater odds (CI 1.39–7.67, *p* < 0.01) of becoming newly food insecure during the COVID-19 pandemic than households without children who were consistently food secure. In addition, participants who lost employment or wages due to the pandemic had 2.51 times greater odds (CI 1.18–5.31, *p* < 0.05) of becoming newly food insecure than participants who had consistent employment and wages who were consistently food secure. 

Various food access-related factors were also significantly associated with becoming newly food insecure during the pandemic in the unadjusted models. Respondents who reported changing their usual shopping location due to food access-related issues had greater odds of identifying as newly food insecure (POR = 2.67, CI 1.26–5.62, *p* < 0.05) than those who did not report going to a new store due to food access-related issues and remained food secure. Additionally, those who sometimes or always had difficulties finding their normal grocery items had 3.83 times greater odds (CI 1.82–8.08, *p* < 0.01) of becoming newly food insecure during the COVID-19 pandemic than those that had little to no difficulties with food availability and were consistently food secure. In addition, households who reported having less than a week of food for their household in their home at the time of the survey had greater odds of becoming newly food insecure (POR = 4.04, CI 1.71–9.53, *p* < 0.01) than those who had a week or more of food in their home at the time of the survey and were consistently food secure.

In the adjusted model, there were also several factors that were associated with greater odds of being consistently food insecure before and during the COVID-19 pandemic. Participants who identified as Latino and/or Black had 4.15 times greater odds (CI 1.63–10.53, *p* < 0.01) of being consistently food insecure than their non-Hispanic white identifying counterparts who were consistently food secure. Households with children had 2.72 times greater odds (CI 1.25–5.91, *p* < 0.05) of being consistently food insecure during the COVID-19 pandemic than households without children who were consistently food secure. In addition, participants who lost employment or wages due to the pandemic had 2.06 times greater odds (CI 1.06–4.04, *p* < 0.05) of being consistently food insecure than participants who had consistent employment and wages and who were consistently food secure.

Food access-related issues were also associated with being consistently food insecure before and during the pandemic. Respondents who reported changing their usual shopping location due to food access-related issues had greater odds of being consistently food insecure (POR = 2.70, CI 1.39–5.25, *p* < 0.01) than those who did not report going to a new store due to food access-related issues and remained food secure. Issues with food availability was also associated with being consistently food insecure, with those who sometimes or always had difficulties finding their normal grocery items had 3.61 times greater odds (CI 1.86–6.99, *p* < 0.01) of being consistently food insecure during the COVID-19 pandemic than those that had little to no difficulties with food availability and remained food secure. In addition, households who reported having less than a week of food for their household in their home at the time of the survey had greater odds of being consistently food insecure (POR = 2.17, CI 1.05–4.49, *p* < 0.05) than those who had a week or more of food in their home at the time of the survey and were consistently food secure.

## 4. Discussion

### 4.1. Summary of Findings

The results from this cross-sectional study showed that various sociodemographic and food access/availability-related factors were associated with being newly food insecure and consistently food insecure during the early part of the COVID-19 pandemic; however, while these factors were similar, they were not identical for both groups. Specifically, households with children, changes in employment and wages, changing shopping locations due to food access-related issues, issues with food availability, and having less than week of food in the home were statistically significantly associated with becoming newly food insecure and being consistently food insecure during the pandemic. Additionally, identifying as Latino and/or Black was additionally associated with being consistently food insecure before and during the COVID-19 pandemic. There have been limited studies in the literature on sociodemographic and food access-related factors associated with food insecurity during the pandemic. However, these findings are consistent with pre-pandemic studies in the literature on food insecurity disparities surrounding income, household composition (presence of children in the home), employment status, race/ethnicity, and issues with food access [28]. Thus, these findings suggest disparities in food insecurity that existed pre-pandemic are being exacerbated and widening due to the COVID-19 pandemic.

### 4.2. Strengths and Limitations of Study

One unique feature of this study is that our sample is diverse, lower income, and with sufficiently large prevalence of food insecurity to allow analysis of changes in food insecurity status from before and during the early part of the COVID-19 pandemic. Additionally, this sample has sufficient sample size and power to employ multinomial logistic regression to examine associations across various sociodemographic and food access-related factors among food secure, newly food insecure, and consistently food insecure households during the pandemic. These findings are from a predominantly lower income, racially/ethnically diverse cohort, representing some of the populations that were the worst hit by the pandemic. Thus, these findings fill a notable gap in the literature.

That said, there are multiple limitations to this study. Most important of all, this was a cross-sectional analysis, precluding us from inferring causality. Second, work on this study was limited to the early stages of the pandemic in Central Texas; it is possible that some of these associations deepened or were otherwise altered as the pandemic progressed. Additionally, there is limited generalizability and external validity due to the geographic specificity and non-representative and snowballed sample. Thus, these findings are more generalizable to groups that are at higher risk for food insecurity (racially/ethnically diverse, lower income, etc.) than for the general U.S. population. Future work at various points during the pandemic could shed greater insight into the experience of food insecurity during later stages of the pandemic. Measures used for this study were relatively simple, and may not have captured all relevant experiences associated with changes in food insecurity. Additionally, future studies should include a more detailed measure of food insecurity and include questions about availability and utilization of food assistance programs in order to analyze this experience with greater nuance. Furthermore, food insecurity status prior to the COVID-19 pandemic was obtained through recall, thus, it may not be the most accurate “pre” measurement. Despite these limitations, the study is a valuable contribution for the reasons noted above.

### 4.3. Public Health Implications and Next Steps

This study provides evidence highlighting that pre-existing social and health disparities have been exacerbated as a result of the COVID-19 pandemic. This rise in food insecurity among under-served populations is a continuing public health issue that needs to be urgently addressed given the association between food insecurity and various health outcomes [14]. Policy and programmatic interventions attempting to mitigate food insecurity during and after the COVID-19 pandemic should strategically target communities and households who were predisposed to food insecurity prior to the pandemic, such as communities of color, low-income communities, communities with limited food access, and households with children. While more studies are needed to determine if our results remain relevant for the later stages of the pandemic in our study setting, and if they are generalizable to other settings, our findings can be used to inform much needed food insecurity prevention and mitigation interventions for under-served communities in Central Texas.

## Figures and Tables

**Table 1:** Demographic characteristics of the sample stratified by food insecurity transition status.

	Stayed Food Secure *n* = 163	Newly Food Insecure *n* = 66	Consistently Food Insecure *n* = 125	Total *n* = 354
	% (*n*)	% (*n*)	% (*n*)	% (*n*)
Race/ethnicity				
Latino and/or Black	41.83% (64)	71.43% (45)	90.08% (109)	64.69% (218)
Non-Hispanic White	58.17% (89)	28.57% (18)	9.92% (12)	35.31% (119)
Presence of children in the household				
No children in household	56.88% (91)	30.30% (20)	21.14% (26)	39.26% (137)
One or more child in household	43.12% (69)	69.70% (46)	78.86% (97)	60.74% (212)
Change in employment status or wages during the COVID-19 pandemic				
No change in employment status or wages during the COVID-19 pandemic	74.03% (114)	39.68% (25)	39.34% (48)	55.16% (187)
Lost job or wages during the COVID-19 pandemic	25.97% (40)	60.32% (38)	60.66% (74)	44.84% (152)
Language spoken at home				
Mainly Spanish spoken at home	19.75% (31)	41.94% (26)	71.90% (87)	42.35% (144)
Mainly English or other language spoken at home	80.25% (126)	58.06% (36)	28.10% (34)	57.65% (196)
Changes in shopping location during the COVID-19 pandemic due to Issues with...				
Availability (usual store did not have the food that was needed), affordability (other stores were cheaper than usual store), and/or accessibility (other stores were closer than usual store)	26.99% (44)	59.09% (39)	59.20% (74)	44.35% (157)
Did not report changing shopping locations due to issues with availability, affordability, and/or accessibility	73.01 (119)	40.91% (27)	40.80% (51)	55.65% (197)
Food availability issues during the COVID-19 pandemic				
Had little to no difficulties finding their normally purchased groceries during the COVID-19 pandemic	64.42% (105)	31.82% (21)	34.40% (43)	47.74% (169)
Sometimes or always had difficulties finding their normally purchased groceries during the COVID-19 pandemic	35.58% (58)	68.18% (45)	65.60% (82)	52.26% (185)
Perceptions of food prices during the COVID-19 pandemic				
Food prices increased during the COVID-19 pandemic	61.35% (100)	71.21% (47)	77.60% (97)	68.93% (244)
Food prices decreased or stayed the same during the COVID-19 pandemic	38.65% (63)	28.79% (19)	22.40% (28)	31.07% (110)
Stability of food supply in household				
Had less than a week of food for the household in their home at time of survey	47.53% (77)	84.85% (56)	80.00% (100)	66.01% (233)
Had a week or more of food for the household in their home at time of survey	52.47% (85)	15.15% (10)	20.00% (25)	33.99% (120)

**Table 2:** Unadjusted and adjusted multinomial logistic regression models examining the association between food insecurity status transitions and sociodemographic and food access-related factors.

Variable (Reference Category for Categorical Variables) *n* = 354	Unadjusted		Adjusted	
	POR (CI)		POR (CI)	
Referent = Was Food Secure Before and During COVID-19	Newly Food Insecure during COVID-19	Was Food Insecure before and during COVID-19	Newly Food Insecure during COVID-19	Was Food Insecure before and during COVID-19
Race/ethnicity				
Latino and/or Black	3.48 (1.84–6.55) **	12.63 (6.41–24.86) **	2.47 (0.97–6.26)	4.15 (1.63–10.53) **
Non-Hispanic White (referent)				
Households with children				
One or more child in household	3.03 (1.65–5.59) **	4.92 (2.88–8.39) **	3.26 (1.39–7.67) **	2.72 (1.25–5.91) *
Households with no children (referent)				
Employment/wage changes due to the COVID-19 pandemic				
Lost job or wages during the COVID-19 pandemic	4.33 (2.33–8.05) **	4.39 (2.63–7.33) **	2.51 (1.18–5.31) *	2.06 (1.06–4.04) *
No employment/wage changes due to the COVID-19 pandemic (referent)				
Language spoken in household				
Mainly Spanish spoken at home	2.94 (1.55–5.56) **	10.40 (5.95–18.17) **	0.59 (0.23–1.52)	2.08 (0.90–4.81)
Non-Spanish speaking at home (referent)				
Changes in shopping location during the COVID-19 pandemic due to issues with…				
Availability (usual store did not have the food that was needed), affordability (other stores were cheaper than usual store), and/or accessibility (other stores were closer than usual store)	3.91 (2.14–7.12) **	3.92 (2.39–6.45) **	2.67 (1.26–5.62) *	2.70 (1.39–5.25) **
Did not report going to a new store because of issues with availability, affordability, or accessibility (referent)				
Food availability issues during the COVID-19 pandemic				
Sometimes or always had difficulties finding their normally purchased groceries during the COVID-19 pandemic	3.88 (2.11–7.13) **	3.45 (2.12–5.63) **	3.83 (1.82–8.08) **	3.61 (1.86–6.99) **
Had little/no difficulties finding normally purchased groceries during the COVID-19 pandemic (referent)				
Perceptions of food prices during the COVID-19 pandemic				
Food prices increased during COVID-19	1.56 (0.84–2.89)	2.18 (1.29–3.69) **	1.16 (0.52–2.57)	1.32 (0.64–2.75)
Food prices did not increase during the COVID-19 pandemic (referent)				
Stability of food supply in household				
Had less than a week of food for the household in their home at time of survey	6.18 (2.95–12.96) **	4.42 (2.58–7.55) **	4.04 (1.71–9.53) **	2.17 (1.05–4.49) *
Had a week or more of food in the home (referent)				

*p* < 0.05 *, *p* < 0.01 **.

## Data Availability

Data can be shared by request and contacting the authors.
